# Frequency of Early-onset Neonatal Sepsis Following Prolonged Rupture of Membranes

**DOI:** 10.7759/cureus.6864

**Published:** 2020-02-04

**Authors:** Heeranand Rathore, Arshalooz J Rahman, Muhammad Salman, Muhammad Nasir, Seharish Sherali

**Affiliations:** 1 Paediatrics and Child Health, The Aga Khan University, Karachi, PAK; 2 Paediatrics and Child Health, The Aga Khan Univeristy, Karachi, PAK; 3 Anaesthesiology, The Aga Khan University, Karachi, PAK

**Keywords:** neonate, sepsis, septicemia, neonatal septicemia, prolonged rupture of membranes, group b streptococcus, preterm

## Abstract

Introduction

In developing countries, sepsis and associated mortality rates in neonatal patients is a serious concern. To improve the outcomes and mortality posed by sepsis, physicians need to know the local epidemiology of the microbial pathogens and their resistance patterns to antimicrobial agents. Therefore, our aim was to determine the frequency of early-onset neonatal sepsis (EONS) following prolonged rupture of membranes (PROM).

Materials and methods

After approval from the ethical review committee, this cross-sectional study was conducted at a tertiary care hospital of a developing country, and informed consent was taken from patients’ parents. All neonates born to a mother with PROM after 24 weeks of gestation up to seven days of life were included. Demographic features, signs of sepsis, blood culture results, and laboratory markers of sepsis were recorded. All data were analyzed by using IBM SPSS Statistics for Windows, Version 20.0 (IBM Corp., Armonk, NY).

Results

A total of 124 patients were enrolled in the study. Seven neonates (5.6%) developed EONS and positive cultures were seen in four neonates (3.2%) with a maternal history of PROM. The organisms identified in cultures were Klebsiella pneumonia, group B streptococcus, Staphylococcus aureus, and Streptococcus species in EONS caused by prolonged PROM.

Conclusions

Early recognition of risk factors, recognition of clinical conditions with prompt laboratory screening for infection, and early establishment of empirical antibiotic treatment are effective preventive measures. Such approaches would be a secure and efficient strategy, particularly in developing countries.

## Introduction

The World Health Organization recently estimated that approximately 130 million infants are born each year globally, of whom four million newborns died at an initial 28 days of life [1]. More than one-quarter of the deaths happened within the first 24 hours, and around three-quarters of the deaths occurred within the first week of neonatal life [1,2]. Neonatal mortality represents 40% of deaths in children younger than five years, globally, and 98% of neonatal mortality occurs in developing countries in the first seven days of life. Every day, nearly 500 newborn babies die in Pakistan [1-3]. Despite recent advances in antimicrobial treatment, neonatal life support measures, and the early detection of risk factors, sepsis continues to be a significant reason behind mortality and morbidity among neonates in developed and developing countries [4]. Neonatal infections presently cause 1.6 million deaths annually and account for 30% to 50% of the overall neonatal mortality annually in developing countries [5]. Among the risk factors, prolonged rupture of the membrane (PROM) is the leading cause of early-onset neonatal sepsis (EONS) and preterm deliveries [6]. Prematurity in neonates and their deficient immunological system make this population more susceptible to infection. The early-onset infective patterns are often vertically transmitted from the mother to the fetus in the perinatal period [7]. Considering the last two decades, most of the relevant studies related to antibiotic resistance of a certain organism showed resistance to first-line antibiotics due to the inappropriate and indiscriminate use of antibiotics, lack of legislation to control antibiotic use, over-the-counter sales of certain antibiotics, and ineffective infection control in the antenatal period [8-10]. Early and appropriate treatment with medicines would also limit the opportunities for extreme complication and mortality along with decreasing the rise of multidrug resistance [5,11]. The selection of the appropriate antibiotic is an ongoing challenge for practicing clinicians due to changing patterns of the microorganisms and their susceptibility patterns [3]. The development of antimicrobial resistance is a great threat. A retrospective study in our population reported the incidence and association of EONS with PROM. We intended to conduct this hospital-based study to determine the current frequency of EONS in neonates born to mothers with PROM. Our objective was to determine the frequency of EONS following PROM.

## Materials and methods

After approval from the ethics review committee, this cross-sectional study was conducted at a tertiary care hospital in a developing country. Informed consent was taken from all patients’ parents. The sample size was calculated as 115 based on the efforts of Bevilacqua et al. [7]. We enrolled 124 neonates to account for chance dropouts by using a consecutive sampling technique. All babies (of both sexes) who were born to mothers with PROM, who were gestational age ≥ 24 weeks at birth on ultrasound and aged up to seven days after birth, were included in the study. Babies whose gestation was shorter than 24 weeks; whose mothers had PROM <18 hours; who received empiric antibiotics before sending septic workup; those discharged home before 48 hours of life; and babies with respiratory distress syndrome, bronchopulmonary dysplasia, transient tachypnea of the newborn, and intraventricular hemorrhage were excluded from the study. Data were recorded on a proforma that documented demographic features, signs of sepsis, blood culture results, and laboratory markers of sepsis. The criteria of EONS were defined according to Alam et al., and PROM was defined as rupture of membrane both artificial and spontaneous before delivery lasting more than 18 hours before labor [3]. The patient’s confidentiality was maintained by standard precautions.

Data analysis

Data were analyzed using IBM SPSS Statistics for Windows, Version 20.0 (IBM Corp., Armonk, NY). Frequency and percentages were computed for categorical variables (i.e., gender and mode of delivery), while the mean and standard deviation were calculated for continuous variables (i.e., birth weight [grams], gestational age [weeks], C-reactive protein [CRP], platelet count, and total leukocyte count). Data were stratified according to gestational age, birth weight, gender, whether the mother received antibiotics before delivery, and mode of delivery for any confounding or effect modification on the outcome. The poststratification chi-square test was applied by taking a p-value <0.05 as significant.

## Results

A total of 124 neonates were included in the study (74 male neonates [59.7%], 50 female neonates [40.4%]). Among all neonates, 45 were preterm (36.3%) and 79 were full-term (63.7%) with the mean gestational age of 36.5 ± 3.3 weeks. Seventy-eight babies were born at an appropriate gestational age (62.9%). The mean birth weight was 2,650 ± 77 grams. A total of 77 neonates (62.1%) were delivered via an emergency lower segment cesarian section. Table 1 describes the demographic features of all neonates. The mean CRP level was 0.74 ± 2.12 mg/dL, mean platelet count was 255.24 ± 69.81 x10^9^/L, and mean total leukocyte count was 12.57 ± 7.12 x10^9^/L. Table 2 describes the descriptive statics of quantitative variables.

**Table 1:** Demographic features of all neonates (N=124) SVD, spontaneous vaginal delivery; LSCS, lower segment cesarean section.

Mode of delivery	
SVD	46 (37.1%)
Elective LSCS	1 (0.8%)
Emergency LSCS	77 (62.1%)
Gender	
Male	74 (59.7%)
Female	50 (40.3%)
Gestational age (weeks)	
Preterm (<37)	45 (36.3%)
Term (37-42)	79 (63.7%)
Birth weight (grams)	
<2,500	44 (35.4%)
≥2,500	80 (64.6%)
Mother received antibiotics	109 (87.9%)

**Table 2:** Descriptive statistics of quantitative variables SD, standard deviation.

Variable	Mean ± SD
Birth weight (grams)	2,650 ± 77
Gestational age (weeks)	36.5 ± 3.3
Total leucocyte count (x10^9^/L)	12.57 ± 7.12
Platelets count (x10^9^/L)	255.24 ± 69.81
C-reactive protein level (mg/dL)	0.74 ± 2.12

EONS occurred in seven (5.6%) neonates, and culture-positive results were seen in four (3.2%) neonates. Stratification of data was done with regard to gestational age, gender, birth weight, mode of delivery, and mother received antibiotics. There was a significant association of EONS with birth weight (p<0.05), as shown in Table 3.

**Table 3:** Stratification with respect to effect modifiers of EONS EONS, early-onset neonatal sepsis; SVD, spontaneous vaginal delivery; LSCS, lower segment cesarean section.

Variable	EONS		p-value
	Yes	No	
Mode of delivery			
SVD	0	46	0.1
Elective LSCS	0	1	
Emergency LSCS	7	70	
Gender			
Male	4	70	0.88
Female	3	47	
Gestational age (weeks)			
Preterm (<37)	3	42	0.71
Term (37-42)	4	75	
Birth weight (grams)			
<2,500	5	39	<0.05
≥2,500	2	78	
Mother received antibiotics			
Yes	5	104	0.16
No	2	13	

Of the seven neonates with EONS, five showed respiratory signs (71.42%), three had tachycardia (42.85%), and one had a fever and poor perfusion (14.8%). Four neonates with EONS showed metabolic acidosis (57.14%), five had elevated CRP levels (71.42%), three had leukocytosis/leucopenia (42.85%), and one had positive findings of pneumonia on chest x-ray (14.28%). Two of the seven mothers of neonates who developed EONS did not receive antibiotics four hours prior to delivery. EONS was significantly associated (p<0.05) with clinical characteristics including fever and poor perfusion, tachycardia and respiratory signs, and laboratory characteristics including metabolic acidosis, elevated CRP, leukocytosis/leucopenia, and positive chest x-ray findings. Of the culture-positive cases, there was one isolate (25%) of each organism including Klebsiella pneumonia, group B streptococcus, Staphylococcus non-aureus, and Streptococcus species.

## Discussion

Neonates are at high risk for sepsis when delivered prematurely by a mother with PROM, and sepsis is the main cause for morbidity and mortality among neonates. The incidence of PROM is 8% to 10% of all pregnancies, and of those, 2% to 4% are associated with intrauterine infection, which places these neonates at risk of sepsis [12]. The incidence of neonatal infection is 1% to 2.6% in infants born from mothers with PROM [13]. Mothers who received antibiotic therapy have a lower risk of transmitting the infection to their newborn babies (4.4%) compared to mothers who have not received antibiotic therapy for PROM (11%) [12]. In our study, we included all neonates who were born of mothers with a history of PROM irrespective of antibiotic intake, and blood culture positivity was detected in 3.2% of the cases. Shah et al. reported that 46% of the neonates who have sepsis were born from a mother with PROM, and another study concluded that PROM is responsible for almost one-third of neonates who develop septicemia [12,14,15]. High chances of neonatal infections are associated with the mother having a group B streptococci infection, PROM lasting longer than 18 hours, maternal fever during labor, and prematurity [14]. The duration of PROM has a significant role in the development of EONS-if PROM persists longer than 24 hours, risks increase from 1% to 5% [16].

Studies in developing countries found that gram-negative organisms remain the major reason for infection in neonates [3,4]. Our study found a similar spectrum of organisms. Bhutta et al. reported that Klebsiella (25%) was the most common cause of EONS in our population, followed by Staphylococcus aureus (14%) and Escherichia coli (8%) [8]. A similar study from a developing country conducted by Joshi et al. concluded that gram-negative sepsis is the leading cause of EONS, occurring in 67.2% of the cases [17]. Among gram-negative species, the Pseudomonas aeruginosa accounts for 38.3% of the EONS cases, followed by Klebsiella (30.4%) and Escherichia coli (15.6%) [17]. Malik et al. reported Klebsiella (55%) as the dominant organism in causing EONS, but Ahmed et al. reported Escherichia coli (30%) was the leading organism causing EONS [9,10]. Alam et al. conducted a retrospective study in our population and found that gram-negative sepsis accounts for 71% of the EONS cases, and Klebsiella was the most common cause [3]. In our prospective cross-sectional study, Klebsiella, Escherichia coli, Staphylococcus aureus, and Streptococcus species were the primary agents causing sepsis, each contributing approximately 25%.

Antimicrobial agents turned out to be significant tools in the treatment of PROM. The strategy of maternal intrapartum antibiotic prophylaxis in high-risk pregnancies has proven extremely effective in the prevention of EONS [18]. Our data suggest that with the current practices in perinatal care, there is variability in the incidence of EONS following PROM. Preventive measures and strategical management should focus on the identification of risk factors for sepsis identification along with prompt laboratory screening therapy based on local institutional data. In developing countries, such approaches would be considered the safest and cost-effective techniques.

## Conclusions

The limited time-span use of antimicrobial agents in neonates, including broad-spectrum antibiotics, to treat disease and prevent colonization improved neonatal sepsis mortality and morbidity. The uncertainty related to the selection of antibiotics can be minimized by the periodic survey of the etiological agent and their antibiotic sensitivity pattern.
